# 

**Published:** 1904-05

**Authors:** 


					﻿NOTICE.— This Journal and the International Journal
of Surgery one year for $1.00 cash, to new subscribers. No
out-of-town checks accepted unless 10c. is added for cost of
cashing. Strictly to new subscribers.
This Journal and Atlanta Semi-weekly Journal one year for $1.25
cash in advance. No checks. Strictly to new subscribers.
NOTICE.—This JOURNAL and THE INTERNATIONAL JOUR-
NAL OF SURGERY one year for $1.00 cash, to new subscribers.
No out-of-town Checks accepted unless 10c. is added for cost
Df cashing.
This Journal and Atlanta Semi-weekly Journal one year for $1.25, cash in ad-
zance. No checks. Strictly to new subscribers.
				

